# The Role of Canonical and Noncanonical Pre-mRNA Splicing in Plant Stress Responses

**DOI:** 10.1155/2013/264314

**Published:** 2012-12-26

**Authors:** A. S. Dubrovina, K. V. Kiselev, Yu. N. Zhuravlev

**Affiliations:** Laboratory of Biotechnology, Institute of Biology and Soil Science, Far East Branch of Russian Academy of Sciences, Vladivostok 690022, Russia

## Abstract

Plants are sessile organisms capable of adapting to various environmental constraints, such as high or low temperatures, drought, soil salinity, or pathogen attack. To survive the unfavorable conditions, plants actively employ pre-mRNA splicing as a mechanism to regulate expression of stress-responsive genes and reprogram intracellular regulatory networks. There is a growing evidence that various stresses strongly affect the frequency and diversity of alternative splicing events in the stress-responsive genes and lead to an increased accumulation of mRNAs containing premature stop codons, which in turn have an impact on plant stress response. A number of studies revealed that some mRNAs involved in plant stress response are spliced counter to the traditional conception of alternative splicing. Such noncanonical mRNA splicing events include *trans*-splicing, intraexonic deletions, or variations affecting multiple exons and often require short direct repeats to occur. The noncanonical alternative splicing, along with common splicing events, targets the spliced transcripts to degradation through nonsense-mediated mRNA decay or leads to translation of truncated proteins. Investigation of the diversity, biological consequences, and mechanisms of the canonical and noncanonical alternative splicing events will help one to identify those transcripts which are promising for using in genetic engineering and selection of stress-tolerant plants.

## 1. Introduction

Being sessile, plants have to adapt to unfavorable environmental conditions via activation of the molecular machinery which increases the chance of plant survival under these conditions. Most adaptations that lead to acquisition of stress tolerance require changes in gene expression, which is regulated transcriptionally and posttranscriptionally [1, 2]. Pre-mRNA splicing, along with mRNA stability, mRNA export from the nucleus, and mRNA translation, are the steps of the important mechanism regulating gene expression at the posttranscriptional level. The multitude and diversity of pre-mRNA splicing events quantitatively and qualitatively affect the mRNA population of a variety of genes. Plant pre-mRNAs, as pre-mRNAs of other eukaryotes, typically include noncoding fragments, introns, which are removed, and coding fragments, exons, which have to be spliced together to form the mature mRNAs. Pre-mRNA splicing is an important step of pre-mRNA processing, which also includes 5′ capping and 3′ polyadenylation. Alternative splicing (AS) is a process when the coding and noncoding fragments of a gene are rearranged in various ways by the spliceosomes at different splice sites, thus generating several mRNA transcripts from the same pre-mRNA molecule (for a recent review of AS mechanisms see [3, 4]). In turn, the mRNA transcripts govern the synthesis of several structurally and functionally distinct protein isoforms, thereby increasing the composition of a plant proteome using a limited number of genes.

Recent genome-wide studies have revealed that AS is highly pervasive in plants. Genome-wide mapping of the *Arabidopsis* transcriptome using the Illumina RNA sequencing (RNA-seq) and high-throughput sequencing of a normalized cDNA library indicated that at least ~42% [5] or ~61% [6] of intron-containing genes in *Arabidopsis* are alternatively spliced, which is considerably higher than previous data (~12–22%) obtained from the cDNA/EST sequencing [7, 8]. Recent studies suggest that environmental stresses can induce AS or alter its efficiency and fidelity in a number of genes playing a role in plant stress response and tolerance [5, 7, 9, 10]. There is also increasing evidence that environmental stresses induce extensive AS, affecting several exons of a mRNA and often lead to the generation of “nonproductive” splice variants of plant stress-inducible gene transcripts [5, 11–13]. These extensive AS events may lead to the production of “nonproductive” transcripts and, thus, decrease gene expression levels or prevent increases in the levels of a full-length protein despite transcriptional stimulation. In addition, there were some studies where noncanonical splicing-like events have been reported for plant genes involved in plant stress signaling pathways. Altogether, the latest data suggest that AS, including noncanonical splicing, might be important in plant stress adaptation. However, despite the phenomenon of AS is known for more than two decades, the list of biological roles for splice variants generated from a single gene in plant stress is not completed and poorly understood. The purpose of this paper is to put together the recent data on AS of plant genes involved in stress signaling and focus on the occurrence, properties, and functional consequences of unconventional splicing and splicing-like events in plants.

## 2. Types and Mechanisms of Pre-mRNA Splicing in Plants

### 2.1. Types and Mechanisms of Canonical AS in Plants

During pre-mRNA splicing, the spliceosome, a large flexible RNA-protein complex, splices out the noncoding sequences and stitches the coding sequences together [14]. Spliceosome consists of several small nuclear ribonucleoprotein particles (snRNPs) and numerous protein factors which recognize specific sequences at the 5′ and 3′ splice sites important in manufacturing the mature RNA. In plants, as in other eukaryotes, the spliceosome recognizes conserved dinucleotides at the ends of introns: the majority of introns start with a GT at the 5′ end, donor site, and end with an AG at the 3′ end, acceptor site [4, 15]. Such labeled introns are removed by the major class U2-type spliceosome. A minority of introns (<1% in *Arabidopsis* and humans), which are removed by the minor U12-type spliceosome, often start with AT and terminate with AC dinucleotides at the 5′ and 3′ ends, respectively. Even more rarely, pre-mRNA splicing occurs at GC–AG or GT–GG donor–acceptor pairs of sites. Altogether, these four pairs of the donor and acceptor sites can be found in the majority of all splicing events and serve as splicing-specific signs. Splice sites in the *Arabidopsis* DNA and DNA of other eukaryotes can be detected using the GeneSplicer Web Interface [16] found at http://www.cbcb.umd.edu/software/GeneSplicer/ or the NetPlantGene Server [17] found at http://www.cbs.dtu.dk/services/NetPGene/. To compile and visualize the evidence for alternative splicing in plants, a comprehensive web-interfaced database ASIP [8] found at http://www.plantgdb.org/ASIP/ was created.

**Figure 1:**
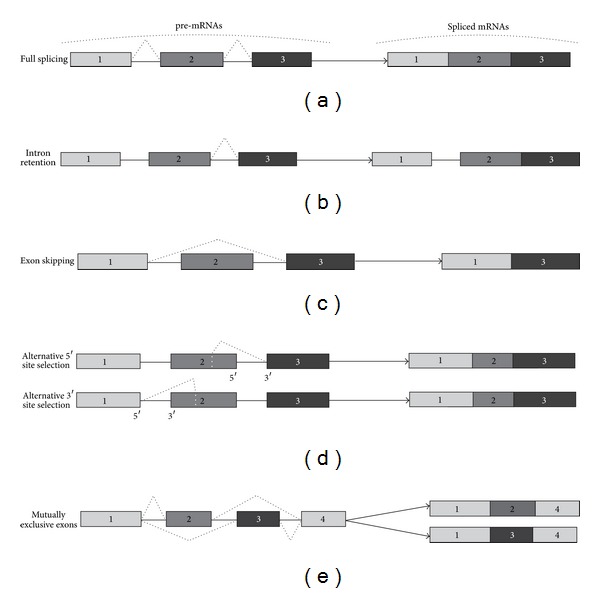
Common types of pre-mRNA splicing in plants. Exons are represented by grey, dark grey and black boxes, and introns-by horizontal solid lines between the boxes. Dashed lines above and below the exons and introns depict AS events. (a) full splicing of a pre-mRNA; (b) intron retention; (c) exon skipping; (d) alternative 5′ or 3′ splice site selection; (e) mutually exclusive exons.

The common types of AS that are generally recognized and are widespread in plants are shown in Figures 1(b), 1(c), 1(d), and 1(e). These AS forms comprise intron retention, exon skipping, alternative 5′ or 3′ site selection, and mutually exclusive exons [4, 18, 19]. It has been generally believed that intron retention (Figure 1(b)) is the most frequent type of AS in plants, representing about 60% of the AS events [8]. However, the latest data reveal that the frequency of intron retention events in the *Arabidopsis* transcriptome is ~40%, while the frequency of AS events that do not include intron retention is ~51% [6]. The authors suggest that the significance of intron retention in generating transcript diversity in plants has been generally overestimated in previous studies.

Such types of AS as exon skipping and mutually exclusive exons (Figures 1(c), and 1(e)) lead to the production of proteins with different rearrangements of their domains or with a deletion of a domain. These rearrangements often result in changed binding properties, activity and stability, and subcellular localization of a protein [4, 19]. Intron retention events and alternative 5′ and 3′ splice sites (Figures 1(b), and 1(d)) more easily lead to the production of mRNAs with premature stop codons (PTCs), which is referred to as “unproductive splicing”. The occurrence of the PTCs may lead either to mRNA degradation via the nonsense-mediated mRNA decay (NMD) mechanism or to the translation of truncated proteins [10, 13]. Besides elimination of the PTC containing aberrant mRNAs, AS coupled to NMD represents a mechanism to control the amount of functional transcripts in the cell through the targeted degradation of specific alternatively spliced isoforms [13, 20], which could have specific biological roles in a living organism. The truncated proteins might be detrimental or energy costly for the cell, and therefore most of PTC carrying mRNAs are eliminated by the NMD machinery before they reached ribosome [10, 21]. However, recent data show that the truncated proteins derived from the PTC containing mRNAs are not necessarily functionless compared to the full-length protein. A number of studies demonstrate that the truncated proteins perform important functions in plant stress adaptation [22, 23].

### 2.2. Types and Possible Mechanisms of Noncanonical AS and AS-Like Events in Plants

**Figure 2:**
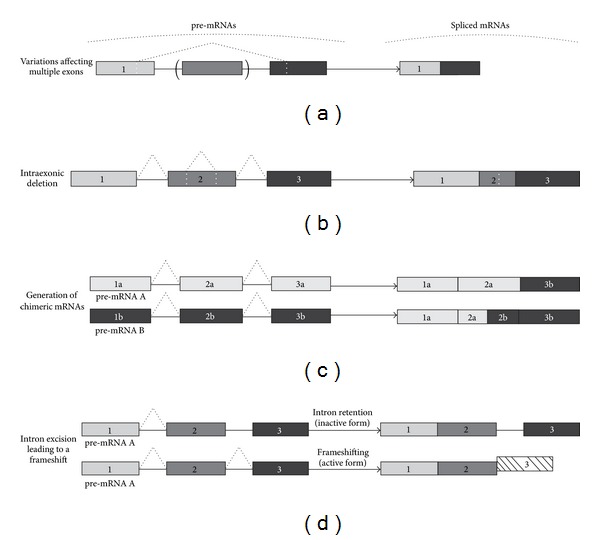
The types of noncanonical pre-mRNA splicing and splicing-like events in plants. (a) variations affecting multiple exons; (b) intra-exonic deletion; (c) generation of chimeric mRNAs; (d) frameshifting as a result of intron excision. Exons are represented by grey, dark grey and black boxes, and introns-by horizontal solid lines between the boxes. Dashed lines above and below the exons and introns depict excision events. The dashed box in part (d) outlines a frameshift caused by an intron excision (details can be found in the Section  3.2). In this case, the spliced mRNA produces a protein with a different amino acid sequence at the C-terminus.

At present, there are several studies, which will be enumerated and discussed in the Section  3.2, where unusual posttranscriptional processing of plant mRNAs has been reported. These noncanonical AS and AS-like events comprised “variations affecting multiple exons”, which refer to deletions of large coding sequences spanning several exons (Figure 2(a)); intraexonic deletions (Figure 2(b)); generation of chimeric mRNAs, which join together portions of two separate mRNA molecules (Figure 2(c)); frameshifting, which was caused by an intron excision (Figure 2(d)). This unusual pre-mRNA processing was often coupled to extensive canonical AS events, including intron retention, exon skipping, or alternative splice site selection and often occurred at noncanonical splice sites.

The noncanonical splice sites were usually represented by short 4–8 nt long direct repeated (SDR) sequences and were different from those splice signals classically recognized by the U2- or U12-type spliceosomes. Recently, Niu et al. [11] identified and analyzed potential SDR-containing sequences in various plant species and described features of the SDRs. They found that the plant SDRs were heavily rich in G and C nucleotides. According to the analysis, GC, CG, CC, and GG dinucleotides were prevalent in the plant SDRs. However, according to the authors, a universal consensus sequence for all the SDRs could not be identified. It has been shown that SDR sequences are indispensable elements for the noncanonical splicing events, essential both for chimeric mRNA transcripts and transcripts with a variety of noncanonical deletions [11, 24, 25].

**Figure 3:**
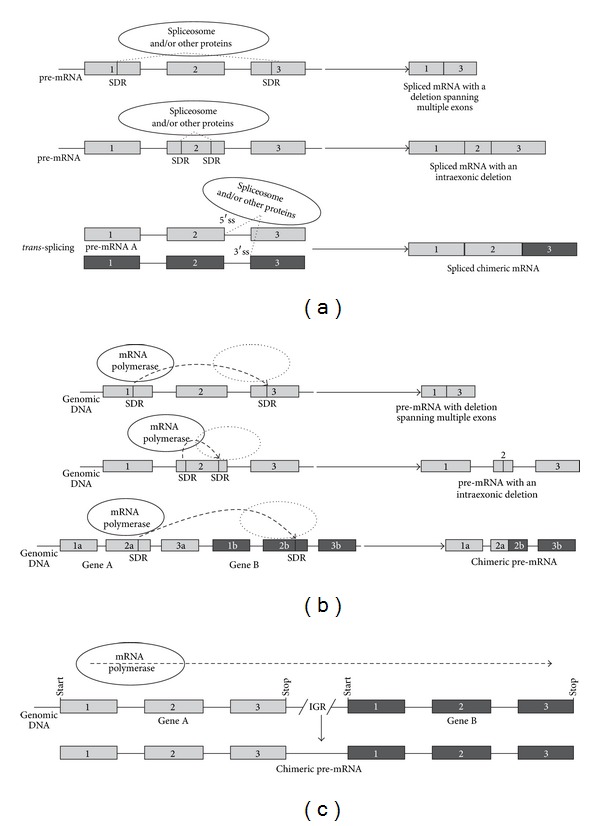
Hypothetical mechanisms of noncanonical pre-mRNA splicing-like events. (a) noncanonical alternative splicing; (b) transcriptional slippage; (c) polymerase read through. Exons are represented by grey or dark grey boxes, and introns—by horizontal solid lines between the boxes. Dashed lines above and below the exons and introns depict AS events. Dashed arrows above the exons and introns depict the direction of RNA polymerase movement. IGR—intergenic region.

The molecular mechanism for generation of the noncanonically processed mRNAs is not known. In case the process involves participation of the spliceosome, such mRNAs could be considered as a product of a noncanonical AS process (Figure 3(a)). However, it is also possible that the noncanonical “alternatively spliced” transcript variants with intraexonic deletions and deletions affecting multiple exons are generated via transcriptional slippage mechanism that require SDR sequences for reannealing of dissociated pre-mRNA molecule from its DNA template strand (Figure 3(b)). In the case of transcriptional slippage, the transcriptional complex (RNA polymerase II and transcription factors), along with the newly synthesized pre-mRNA, could dissociate from its DNA template during transcription and subsequently reassociate at an identical SDR sequence. Ritz et al. [26] suggested that this mechanism was used for generation of intraexonic deletions and deletions affecting multiple exons, which have been discovered in their study in the human SGCE gene, associated with the neurological movement disorder myoclonus dystonia. Ritz et al. [26] supported their data by analyzing SGCE gene expression in zebrafish, rats, and mice. The noncanonical splicing-like events, which were discovered in the plant stress-inducible genes and are described in the Section  3.2, exhibit similar features to the noncanonical transcript variants described by Ritz et al. [26] and, therefore, could be also generated by the intramolecular slippage mechanism.

A number of mechanisms have been suggested to produce chimeric mRNA transcripts: (1) *trans-*splicing (Figure 3(a); [27]), when two different mRNAs are spliced together by the spliceosome and, presumably, some other *trans-*acting protein factors participate in this process; (2) the read-through mechanism (Figure 3(c); [28]), when the RNA polymerase transcribes two adjacent genes by reading through the intergenic DNA sequence; (3) the transcriptional slippage mechanism (Figure 3(b); [25]), when the RNA polymerase dissociates from SDR sequences on the DNA template with subsequent reassociation at a homologous SDR. It has been suggested that mRNA fusion transcripts with canonical splice signals are spliced via the *trans*-splicing mechanism, while fusion transcripts lacking canonical splice sites and harboring SDRs could be spliced via the transcriptional slippage [25, 26].

It is reasonable to hypothesize that the dissociation and subsequent association of the newly transcribed mRNAs with the DNA template, that is, transcriptional slippage, are not spontaneous and depend on environmental conditions. Ritz et al. [26] suggested that, in stress situations, transcriptional regulation becomes less strict, and this results in enhanced transcriptional slippage and accumulation of the low frequency noncanonical splicing-like transcript variants.

## 3. The Role of AS in Plant Stress Response

### 3.1. Canonical AS and Plant Stress Response

A number of AS events have been reported to occur in plants in response to biotic and abiotic stresses. Genome-wide studies demonstrate that various environmental stresses change AS profiles and increase the number of alternatively spliced transcripts in *Arabidopsis* [5, 7]. Some of the alternatively spliced transcripts were induced in response to biotic stresses and were shown to encode regulatory proteins with important functions in plant pathogen response pathways. These regulatory genes, whose mRNAs are alternatively spliced in response to pathogen attack, include pathogenesis-related (PR) genes, such as chitinase [29]; defense-related genes, such as isochorismate synthase [30]; genes encoding cyclotides-defence peptides with insecticidal and antimicrobial activities [31]; the plant resistance (R) genes encoding nucleotide-binding site-leucine-rich repeat (NBS-LRR) proteins, which are responsible for the intracellular detection of pathogen-related elicitors [22, 32, 33].

In response to abiotic stresses, AS also affects a range of regulatory genes that are presumed to function in abiotic stress adaptation of plants. These genes include mitogen-activated protein kinases (MAPKs) [34–36]; several groups of stress-related transcription factors, for example, MYB and CBF/DREB transcription factors [23, 37–39]; ubiquitin ligases and transcription factors, which are considered to be involved in protein degradation in response to environmental cues [40, 41]; the serine/arginine (SR) proteins [42, 43], which are important splicing regulators in the plant development and stress responses [44]. The examples of AS events concerning regulatory genes involved in biotic and abiotic stress response and mentioned above were enumerated and discussed in detail in a recent review by Mastrangelo et al. [10].

Some other gene families playing important roles in plant adaptation to environmental stresses are reported to be alternatively spliced in plant cells, for example, dehydrin genes in *Vitis riparia* and *V*. *vinifera* [45], whose protein products accumulate in response to various dehydrating stress conditions; potato invertase genes, which are responsible for cold-induced potato sweetening [46]; Δ1-pyrroline-5-carboxylate synthetase1 (*P5CS1*) gene, whose AS contributes to the natural variation in proline accumulation between different *Arabidopsis* accessories [47]; the genes of JASMONATE ZIM-domain (JAZ) proteins, which regulate jasmonic acid signaling in response to biotic stress [48]; the genes of wound-responsive RNA-binding UBA2 proteins [49]; the L-myo-inositol-phosphate synthase (*MIPS*) genes, induced in response to a variety of abiotic stresses [50]; the rice *Sultr* genes encoding sulphate transporters, which regulate sulphur status during stress conditions [51].

A considerable part of the AS events resulted in the occurrence of PTC carrying mRNAs which cannot not be translated into full-length proteins. As it is currently believed, such mRNAs can be either degraded by the NMD machinery or translated into truncated proteins lacking some active domains [10]. There is growing evidence that AS coupled to NMD not only serves to eliminate aberrant mRNAs but also plays a certain role in plant stress response (see Section 4 of the present review). There are several studies demonstrating important biological role of truncated proteins in plant stress adaptation. For example, the transcripts of plant R genes are alternatively spliced to produce truncated protein forms with different combinations of functional domains whose existence has been confirmed by Western blotting and other methods [22, 32], and it has been shown that the truncated proteins induce hypersensitive response-like cell death and, thus, help plants combat bacterial pathogens [22]. Special attention attracts the report by Matsukura et al. [23] that truncated nonfunctional transcription factors OsDREB2 were generated in the absence of stress in rice; however, when stress conditions were applied, the functional full-length *OsDREB2* transcript was generated via stress-inducible alternative pre-mRNA splicing and then performed its function to activate expression of stress-responsive genes. Thus, it is possible that generation of a nonfunctional protein in the absence of stress allows plants to avoid the detrimental effects and high metabolic cost for producing the proteins conferring resistance to biotic and abiotic stresses. Mastrangelo et al. [10] also suggest that generation of a full-length protein in response to stress by changing splicing pattern of the corresponding gene could prevent spending time on transcriptional activation and accumulation of the necessary mRNAs.

A number of studies describe extensive AS in plant genes when splicing events affected multiple exons in response to environmental stress. The phenomenon of extensive missplicing in plant genes has been first described for the In1 and Vp1 transcription factors in maize and wheat, respectively [52, 53]. R1/b1 transcription factors, including In1 transcription factor, control anthocyanin biosynthesis, which have been shown to protect plant tissues from photoinhibition, or high-light stress [54]. The In1 transcription factor encodes a repressor of anthocyanin biosynthesis in maize seeds [52]. The missplicing events resulted in the production of a nonfunctional In1 protein. This might be a mechanism ensuring that normal anthocyanin production was not suppressed. McKibbin et al. [53] demonstrated that extensive missplicing of wheat Vp1 transcription factor transcripts contributes to the susceptibility of wheat to an environmentally triggered disorder, preharvest sprouting. The disorder occurs when grains mature under cool and damp conditions. There was a variety of deletions of different length in several exons of the gene which resulted in the loss of a functional domain, introduction of PTCs, and shifted reading frame.

Filichkin and Mockler [12] demonstrated that extensive unproductive AS is a widespread phenomenon, which frequently generates mRNA isoforms harboring in-frame PTCs, among plant circadian clock genes. Plant circadian clock and abiotic stress are regarded as firmly interconnected processes [55]. Filichkin and Mockler [12] reported that the relative ratios of the PTC carrying mRNAs encoding for several key circadian clock regulators can be considerably shifted under abiotic stress conditions. In particular, their results indicate a potential role of extensive AS and nonsense transcripts of the CCA1/LHY-like subfamily of MYB transcription factors in the regulation of circadian rhythms. According to Yuan et al. [30], isochorismate synthase of *Populus trichocarpa* encoded by a single gene undergoes extensive AS, and this results in the generation of at least 37 splice variants where intron retention and alternative acceptor splice sites predominated as AS types. A significant portion of the transcripts was generated by several types of AS events and resulted in the loss of multiple exons. Similarly to the phenomenon described by Filichkin and Mockler [12], the majority of the transcripts contained PTCs and might be targeted for degradation via NMD.

Taken together, these data indicate that AS does not represent “noise” of the cellular stress. It is rather a mechanism playing an important role in plant adaptation to unfavorable environmental conditions through degradation of specific mRNA transcripts or generation of new truncated protein forms. This class of events can be classified as expansion of molecular basis for search of new ways of adaptations. The effect of AS on plant stress response is probably still underestimated, and a number of not yet known AS-based mechanisms are likely to play a role in the plant adaptation to adverse environmental conditions.

### 3.2. Noncanonical AS and AS-Like Events in Plant Stress Response

There are several studies where extensive missplicing of plant stress-responsive gene products has been reported [11, 24, 56–61]. The missplicing events often included intron retention; exon skipping, alternative 5′ and 3′ splice sites selection (Figures 1(b), 1(c), and 1(d)) and were coupled to the occurrence of multiple noncanonical pre-mRNA processing events, which resembled alternative splicing (Figures 2(a), 2(b), and 2(c)).

Several cases of such unusual posttranscriptional pre-mRNA processing have been recently reported for choline monooxygenase (CMO) and betaine aldehyde dehydrogenase (BADH) genes in rice [24, 56]. The SDR-mediated unusual processing of *BADH* transcripts was also detected in other cereal crops, including maize (*Zea mays*), wheat (*Triticum aestivum*), and barley (*Hordeum vulgare*) [56]. CMO and BADH are responsible for glycine betaine (GB) synthesis in plants, which are capable of synthesizing this compound, so-called GB accumulators [62]. GB, a quaternary ammonium solute, is a metabolite playing a crucial role in developing osmotic tolerance [63]. Rice is defined as non-GB accumulator, since there is no evidence of detectable GB accumulation in rice plants [64]. Luo et al. and Niu et al. [24, 56] have shown that most rice *CMO* and *BADH* transcripts are processed incorrectly, generating transcripts with retained introns, with a variety of deletions of coding sequences, or with insertions of exogenous gene sequences. The unusual deletion events occurred at the SDR sequences which were present at 5′ and 3′ splicing junctions that were distinct from conventional (U2/U12-type) splicing boundaries.

Luo et al. and Niu et al. [24, 56] proposed that the SDRs are required for the recognition of the deletion sites in response to stress conditions. The unusual SDR-mediated posttranscriptional processing events included partial exon deletions, exon fragment repetitions, deletions affecting multiple exons (Figure 2(a)), intraexonic deletions (Figure 2(b)), and generation of chimeric mRNAs (Figure 2(c)), which resulted in either partial loss of an exon or unusual exonic sequence rearrangements within or between RNA molecules. These noncanonical mRNA modifications were coupled to extensive AS events of common types, such as intron retention or selective exon inclusion (Figures 1(b) and 1(e)). According to Niu et al. [56], the site selection for the deletions/insertions was altered in response to the stress conditions. At the protein level, canonical and noncanonical splicing events resulted in the removal of the translation initiation codon, deletion of a functional domain, and frameshifts with premature termination of translation by introducing PTCs. Taken together, these findings indicate that a lack of precise *CMO* and *BADH* mRNA transcripts contributes to the variation of GB-synthesizing capacities among various plant species and the absence of GB in rice [24, 56]. In support of the data, Fan et al. [57] described similar extensive missplicing of the rice VP1 transcription factors which contribute to the development of stress-mediated preharvest sprouting disease in rice. The SDR sequences were again present at the junctions of the unusual excision sites in the *VP1* splice variants.

Using the GenBank database, Niu et al. [11] predicted that 24 plant candidate genes involved in diverse functional pathways and belonging to both monocots (wheat, barley, and maize) and dicots (*Arabidopsis* and tobacco) potentially possess SDR-mediated posttranscriptional processing. These potential SDR-mediated processing events were experimentally detected by the authors using the known SDR-containing sequences as probes for RT-PCR and subsequent DNA sequencing. The authors also demonstrated that the paired presence of SDRs is necessary but not sufficient in SDR-mediated splicing in transient and stable transformation systems.

The sunflower *sf21C* gene is a member of a small plant gene family related to the human N-myc downstream-regulated gene family (NDRG), which is involved in stress and hormone responses [65, 66]. Lazarescu et al. [58] characterized 20 splice isoforms of the *sf21C* gene and reported that five identified variants were generated by splicing at novel splice sites, different from those classically recognized by U2- and U12-type spliceosomes. These noncanonical splice sites were again represented by the SDR sequences. In addition, twelve transcript variants contained PTCs, which resulted in frameshifts and possible translation of truncated proteins of different length and structure. Although the *sf21C* transcripts with PTCs are potential candidates for NMD, they were abundant and occurred in distinct combinations in some sunflower organs and, therefore, the transcripts could be translated into truncated proteins and exert a certain biological function. The authors suggest that the structural modifications of the proteins may affect their compartmentalization (nucleus or other cell organelles) or binding properties.

Zou et al. [59] described a similar case of extensive AS with noncanonical splice variants when studying the structure of the prolyl 4-hydroxylase (*P4H*s) genes and their expression patterns in maize seedlings under waterlogging. In plants, P4Hs are suggested to function as oxygen sensors under hypoxia stress [67, 68]. Zou et al. [59] found that *ZmP4H* genes displayed different expression patterns under waterlogging, and many *P4H* transcripts were spliced using nonconventional splicing sites at the exon/intron junctions containing SDRs. Many of the *P4H* splice variants contained PTCs. The authors propose that the splicing events might be important in the regulation of *ZmP4H* genes under waterlogging stress in plants.

A new type of a noncanonical intron, again with SDR sequences at 5′ and 3′ splice sites, was found in some of the alternatively spliced transcript variants of the R2R3-MYB transcription factor in *Arabidopsis* and rice [60]. Thirty-eight *Arabidopsis* and rice genes were found to have this type of noncanonical intron, and most of the genes were suggested to undergo AS. In addition, a noncanonical intron flanked by SDRs has been reported for *FCA* [61], an *Arabidopsis* gene controlling flowering time, which is a stress-dependent event. The authors detected four transcript variants of the *FCA* gene. Transcript *δ*, representing approximately 10% of the *FCA* transcripts, was alternatively spliced at one of its introns which was flanked by two 6 bp repeated sequences. The authors also noted that neither of the repeats incorporates canonical splice sites.

It is well established that protein folding in the endoplasmic reticulum (ER) is disturbed in plants upon environmental stress [69]. The Unfolded Protein Response (UPR) is a cellular stress response mechanism which is activated in response to the accumulation of unfolded or misfolded proteins in the lumen of ER and is aimed to alleviate the ER stress by arresting protein translation and activating signaling pathways to increase the production of molecular chaperones [70, 71]. It has recently been established that in response to the ER stress the IRE1 kinase catalyzes unconventional splicing of the bZIP60 transcription factor to produce active form of the protein in *Arabidopsis thaliana* [72]. The active bZIP60 transcription factor moves to the nucleus and activates transcription of the genes responsible for the UPR. During the ER stress response, a 23 nt intron is spliced out from the *bZIP60* mRNA (Figure 2(d)). The intron excision leads to a frameshift and a PTC (Figure 2(d)), and this, in turn, generates a different amino acid sequence in the C-terminal region of the protein. Due to the splicing event, the bZIP60 protein loses the domain that anchors it on the ER membrane. The orthologue of the bZIB60 transcription factor in *Oryza sativa*, OsbZIP74 (also known as OsbZIP50), has been shown to be similarly activated by the unconventional splicing leading to a frameshift [73, 74].

Currently, there is accumulating evidence that *trans-*splicing is a mechanism of modification of gene expression in mammals, flies, and nematodes [27, 75], which is to a certain part analogous to horizontal gene transfer, considering that it occurs at the molecular level and in a single cell. In a recent study, Zhang et al. [76] demonstrated the high complexity of the rice transcriptome using deep RNA sequencing at single base-pair resolution. Unexpectedly, the authors identified 234 putative chimeric transcripts (Figure 2(c)), containing parts of two different genes, which seem to be produced by *trans*-splicing (Figure 3(a)). However, there is also a likelihood that chimeric mRNAs might be produced by a mechanism that does not involve participation of a spliceosome, for example, via transcriptional slippage (Figure 3(b)) or read-through transcription (Figure 3(c)).

Although there is compelling evidence for a relatively high number of *trans*-splicing events in mammals, flies, and nematodes [27, 75], there is scarce information on the occurrence of fused transcripts of such origin in plants. To our knowledge, there is clear evidence for only two *trans*-splicing events observed during transcription of plant genes. Kawasaki et al. [77] demonstrated that two RNAs were independently transcribed from the *SPK-A* and *SPK-B* genes of *O*. *sativa* and joined in a chimeric mRNA, possibly by *trans*-splicing. The rice *SPK* genes encode calcium-dependent protein kinases (CDPKs), which are known to play an important role in mediating plant biotic and abiotic stress responses [78]. He et al. [79] have found and provided evidence that the nodule specific *MsHSF1c* transcript of a heat-shock transcription factor may be generated by *trans*-splicing mechanism. In addition, in our research, we identified extensive noncanonical splicing-like events, including intraexonic deletions, an insertion of extraneous 35 bp sequence of unknown origin in the kinase domain of grape *CDPKs*, or generation of chimeric *CDPK* transcripts, when analyzing expression of the *CDPK* genes in cell cultures of *V*. *amurensis* and Panax* ginseng* with different levels of tolerance to salt stress [80, 81]. Similar posttranscriptional modifications were also observed for *CDPK* genes in somatic embryos of *P. ginseng* [82, 83]. Brummell et al. [84] provided evidence for the generation of chimeric transcripts encoding potato invertase inhibitors and their active accumulation in response to cold stress. The potato invertase inhibitors are known to prevent invertase-induced hexose accumulation in potato tubers (potato sweetening) in response to low temperatures. However, *trans*-splicing is probably not the case for the chimeric mRNAs of the invertase inhibitor, since these mRNAs are generated using noncanonical splice sites represented by SDRs.

In summary, these experimental data suggest the existence of molecular mechanisms that do not follow conventional notion of splice site selection in plant AS and indicate the involvement of the noncanonical splicing and splicing-like events in plant stress responses. Further research is needed to uncover mechanistic consequences of the presence of SDRs in a variety of plant stress-related genes and physiological roles of the SDR-mediated noncanonical splicing-like events.

## 4. The Role of AS Coupled to NMD in Plant Stress Response

NMD surveillance machinery is a mechanism that identifies cellular mRNAs carrying PTCs and targets these mRNAs for degradation, thereby preventing accumulation of truncated proteins, which probably could have deleterious effects on the cell metabolism (for recent reviews on NMD mechanisms, see [85–87]. Briefly, NMD is triggered by exon junction complexes (EJCs; components of the assembled ribonucleoprotein particles) that act during the mRNA splicing process and recruit the key NMD *trans*-acting protein factors, UPF proteins, thereby leading to formation of functional NMD complexes and rapid degradation of the PTC-containing mRNA. In plants, both introns and long 3′-UTRs operate as *cis*-acting elements, which are recognized by the *trans*-acting NMD factors. NMD is a widespread phenomenon and is conserved among different eukaryotes, including plants [21]. Genome-wide studies revealed that up to 20–30% of the *Human*,* Drosophila*, and *Caenorhabditis* mRNA transcripts can be targeted to degradation via NMD [88–90]. Surprisingly, according to a recent genome-wide mapping of AS in *A*. *thaliana* [5, 13], alternatively spliced transcript isoforms with PTCs comprised the majority (~45–78%) of alternatively spliced transcripts in the analyzed *A*. *thaliana* transcriptome. About 13–18% of intron-containing genes are potentially regulated by AS coupled to NMD [13]. Although little is known about the functional roles of NMD in plants, there is increasing evidence that AS coupled to the NMD-mediated mRNA degradation not only removes aberrant mRNAs but also acts as a mechanism to regulate gene expression, including expression of genes playing essential roles in plant stress adaptation.

Filichkin et al. [5] demonstrated that the relative ratios of the PTC containing mRNAs can be significantly shifted for several key regulatory genes under abiotic stress treatments in *A*. *thaliana*. At present, a number of genes known to be involved in stress response pathways in plants have been shown to undergo AS coupled to NMD. Palusa and Reddy [91] provided evidence that a significant part of PTC containing splice variants of the SR proteins, which are known to regulate plant AS in response to environmental stress, are subject to degradation by the NMD mechanism. Sugio et al. [92] showed that the specific heat shock factors (HSFs), notably HSF2A, are also regulated by AS coupled to NMD. NMD-deficient *Arabidopsis* mutants were shown to possess constitutive pathogenesis-related (PR) gene expression, salicylic acid (SA) accumulation, and increased resistance to pathogens [93]. Furthermore, a recent study by Rayson et al. [94] has found that the majority of NMD-targeted transcripts in *A*. *thaliana* mutants deficient in NMD are associated with response to pathogens. Those NMD mutants, where the NMD-targeted transcripts were elevated, exhibited partial resistance to *Pseudomonas syringae*. This finding indicates that gene expression regulation via AS coupled to NMD possesses specific biological roles. Rayson et al. [94] proposed that plants may employ NMD-controlled gene expression as a means to coordinate pathogen responses. The findings by Rayson et al. [94] suggest that understanding the biological consequences of AS coupled to NMD is prospective and necessary for developing new approaches in crop protection.

In a recent genome-wide study, Kalyna et al. [13] showed that AS coupled to NMD affects the transcript abundance and thus regulates expression of many stress-responsive genes in *A*. *thaliana*, including transcription factors (e.g., At-Di19-5 and Zn finger B-box type protein), protein kinases (e.g., CRK18, CPK28, the SNF1-like protein kinase, and AtKIN11YY), calcium sensors (e.g., SOS2 and SOS3/CBL4), various temperature, drought and salt response factors (e.g., SRF2 and HSF2A), and some other genes involved in stress adaptation. Unexpectedly, Kalyna et al. [13] have also found that a considerable part of the alternatively spliced transcripts retaining introns, which are known to be the most common AS type in plants [5, 6], do not trigger NMD even though they possess PTCs and other classical NMD-inducing features. Thus, the question of the destiny and biological roles of the PTC containing transcripts in plant stress response and other cellular processes remains open. It is highly likely that the intron-retaining transcripts, which are not targeted to NMD, are translated into truncated proteins playing essential roles in plant development, metabolism, and stress adaptation.

There is compelling evidence that AS coupled to NMD regulates expression of plant genes involved in plant stress response. Although the precise roles for the phenomenon are not known, it appears that unproductive, in terms of protein translation, AS is exploited by plants to adapt to changing environmental conditions. Further research is needed to clarify the involvement of AS coupled to NMD in plant stress responses.

## 5. Rare Noncanonical Transcriptional Events or Artifactual RT-PCR “Splicing”?

It has long been known that during reverse transcription of the retroviral genome, reverse transcriptase makes a number of template switches in a homology-dependent manner, which is necessary for the retroviral viability and variability [95, 96]. During reverse transcriptase template switching, the growing cDNA strand can dissociate from the RNA template and reassociate with a homologous region on the same RNA template (intramolecular switching) or with a homologous region on a different RNA template (intermolecular switching) thus implementing the *cis-* and *trans-*events, respectively. The homologous regions are usually represented by short 8 nt long direct repeats. Not surprisingly, during cDNA synthesis *in vitro *reverse transcriptase can also switch from one template to another and, thereby, generate artificially deleted or chimeric cDNA transcripts [96–99]. Thus, during intramolecular reverse transcriptase template switching, cDNAs bearing artificial deletions are generated [97]. These cDNAs can be misinterpreted as alternatively spliced mRNA transcripts. Intermolecular template switching often results in the synthesis of artificial chimeric cDNAs, which can be misinterpreted as *trans*-spliced mRNAs [99].

A number of studies provided clear evidence that some deleted or chimeric cDNAs, previously identified as alternatively spliced or *trans*-spliced, are artifacts of reverse transcription and are often present in cDNA databases [97, 99–103]. For example, Cocquet et al. [97] searched for the template-switching artifacts in cDNA databases by scanning a collection of human splice sites (Information for the Coordinates of Exons, ICE database). The authors discovered that artifacts represent a significant portion of the noncanonical “spliced” transcripts deposited in the database. These artifactual transcripts did not possess canonical splice signals and contained SDR sequences instead, which indicates that these cDNAs could be examples of the reverse transcriptase switching. According to Cocquet et al. [97], the template-switching events were also present, but rare, among transcripts with canonical splice sites. The authors experimentally confirmed several cases of the putative template switching events. The observations by Delviks and Pathak [104], Mader et al. [101], and Cocquet et al. [97] demonstrate that long sequences between the SDRs and the propensity of the sequences to form stem-loop secondary structures both increase the probability of onset of template-switching events.

Houseley and Tollervey [99] recommend applying methods which do not include reverse transcription in order to determine whether a truncated or chimeric transcript is a true splicing event or an artifact. *In vitro* transcription, northern blotting or RNase protection assay can be used for this purpose [97, 99]. Also, it is possible to use a reverse transcriptase enzyme with increased thermal stability, since the reverse transcriptase template switching is usually not detected anymore in this case [97, 99]. However, according to Houseley and Tollervey [99], using a heat-stable reverse transcriptase does not completely eliminate the probability of reverse transcriptase template switching.

Although there is compelling evidence for the capability of reverse transcriptase to generate artifactual splicing-like products *in vitro*, it appears reasonable to admit that there is also likelihood that the noncanonical splicing-like events in the plant stress-responsive genes are generated via the transcriptional slippage mechanism or a noncanonical AS process *in vivo*. In case of transcriptional slippage, RNA polymerase might dissociate from the DNA template *in vivo* similarly to reverse transcriptase. Support for this assumption may be found in a recent genome-wide study by Ritz et al. [26] demonstrating that the transcriptional regulation is much more complex than previously believed and that the rare unusual splicing events, including deletions affecting parts of adjacent exons, intraexonic deletions, and chimeric transcripts, were not artifacts of PCR, sequencing, or reverse transcription. The authors provided evidence for the existence of such noncanonical transcripts not only in the human tissue but also in mouse, rat, and zebrafish. They proposed that the transcript variants were generated as a result of intramolecular slippage. Recently, Zhang et al. [76] conducted a comprehensive study of the rice transcriptome using high-throughput paired-end RNA-seq and found a large number of chimeric transcripts which appeared to arise as a result of *trans*-splicing. The chimeric RNAs showed partial alignment to two genes, which often are present on different chromosomes. Unfortunately, the RNA-seq approach again employs reverse transcription to prepare cDNA library and sequence a transcriptome, and therefore care should be taken when arriving at a conclusion on the RNA-seq results. Nevertheless, several cases of the unconventional splicing-like events using noncanonical splice signals in the plant stress-related genes were verified using such alternatives to RT-PCR as RNase protection assay or *in vitro* transcription (e.g., [61, 68, 77, 79]).

Since the noncanonical AS events in the plant stress-responsive genes in the majority of experiments have been identified using RT-PCR and subsequent sequencing of the amplified RT-PCR products (e.g., [11, 24, 56, 58, 59]), there is likelihood that at least some of the reported alternatively spliced transcripts were artifacts of reverse transcription. It is particularly likely for the noncanonical splicing events, where SDRs are present instead of the canonical splice signals. Thus, one should admit that the phenomenon of reverse transcriptase template switching may hamper the identification of truly alternatively spliced mRNAs, and it raises the question of what portion of the identified noncanonical splicing and splicing-like events in plant stress-related genes is genuine.

Numerous stipulations of authors of primary investigations, which we conserved deliberately in our review, seem to cast doubt on the very existence of such complex events in plants and other higher organisms. However, the validity of search in this direction is based on the following reasons.

- The first and most general reason arises from the notion about the hierarchical self-similarity of biological world. As far as we know, the mixing and rearrangement of genetic material are key events in living systems development (see, e.g., [105–107]), so their occurrence can be expected at the all levels of presentation of biological world. Indeed, symbiogenesis and horizontal gene transfer events correspond to taxon level of presentation and sex reproduction, chromosome pairing, and crossover-to the organismal, chromosomal, and gene levels, respectively. The life is very sophisticated in its invention of new devices and representations: a recently discovered human gut microbiome with its collective gene set is a good example. So the mixing of more small fragments of genetic material (including mixing and rearrangement via *cis*- and *trans*-splicing) seems reasonable to correspond to subgene level.
- The NMD mechanism occurrence in higher organisms is well proved, and this fact supposes the availability of substrate for its operation. Noncanonical splicing can supply the NMD mechanism with this substrate.
- The results of *in vitro* experiments (where chimeric cDNAs synthesized by switching of reverse transcriptase were similar to splicing products) compel one to search the analogy in native cell. The full analogy seems to be impossible since there is no clear evidence of significant activity of reverse transcriptase in the healthy plant cells. However, some quantities of extragenomic DNA can appear supposedly in a plant cell in some way, for instance, in a way similar to that in which the fetus DNA has appeared in maternal plasma [108]. This extra DNA being modified in aggressive surroundings could serve as matrices for synthesis of unusual transcripts. However, the plasma system analog in plants, apoplast, represents an effective barrier for long-distance DNA transport [109]. The mobile small RNAs can move from cell to cell inducing gene silencing and some other effects [110], but these molecules are too small to imitate the products of noncanonical splicing. So, the splicing hypothesis is the simplest for *in vivo* context.
- Finally, the unusual transcripts were detected in plant cell, and their contents were shown to depend on the physiological state of cells. This dependence is in agreement with logics about the behavior of plant in the stress conditions, whereas no stress correlations can be expected in the case of method errors.

## 6. Conclusion

On reviewing the literature it may be stated that environmental stresses have an impact on AS, which, in turn, affects plant stress responses and might promote plant stress tolerance to adverse environmental conditions. AS enriches the response capacity of cells by enabling them to synthesize structurally and functionally different proteins from a gene and contributes to the complexity of both transcriptome and proteome to increase the survival potential of a phenotype in various physiological conditions. Much remains to be discovered about the molecular mechanisms of the various AS and AS-like events that increase the plasticity of plant transcriptome and proteome in such a tremendous rate. In addition, it is intriguing why plants spend so much energy to produce numerous “unproductive” splice variants containing PTCs and then just “kill” them in the recently discovered NMD degradation pathway. This might represent a still obscure but important evolutionary strategy of living to substitute in parts the natural selection of phenotypes by molecular selection of genetic elements. Studies of the transcript diversity generated in response to environmental stresses as well as uncovering-specific biological consequences of some principal splicing events are perspective for plant biotechnology in terms of developing new strategies for crop breeding and protection.

## Abbreviations

AS: Alternative splicing
BADH: Betaine aldehyde dehydrogenase
CMO: Choline monooxygenase
GB: Glycine betaine
ORF: Open reading frame
NMD: Nonsense-mediated mRNA decay
pre-mRNAs: Intron-containing precursor-mRNAs
SDRs: Short direct repeated sequences
PTCs: Premature stop codons
RNA-seq: Illumina RNA sequencing.
